# Survival or death: a dual role for autophagy in stress-induced pericyte loss in diabetic retinopathy

**DOI:** 10.1007/s00125-016-4058-5

**Published:** 2016-07-30

**Authors:** Dongxu Fu, Jeremy Y. Yu, Shihe Yang, Mingyuan Wu, Samar M. Hammad, Anna R. Connell, Mei Du, Junping Chen, Timothy J. Lyons

**Affiliations:** 1 Centre for Experimental Medicine, School of Medicine, Dentistry and Biomedical Sciences, Queen’s University Belfast, 97 Lisburn Road, Belfast, BT9 7BL UK; 2 Department of Immunology, Harbin Medical University, Harbin, People’s Republic of China; 3 Section of Endocrinology and Diabetes, University of Oklahoma Health Sciences Center, Oklahoma City, OK USA; 4 Department of Regenerative Medicine and Cell Biology, Medical University of South Carolina, Charleston, SC USA

**Keywords:** Apoptosis, Autophagy, Diabetic retinopathy, ER stress, LC3B, Modified LDL, Oxidative stress, Pericytes

## Abstract

**Aims/hypothesis:**

Intra-retinal extravasation and modification of LDL have been implicated in diabetic retinopathy: autophagy may mediate these effects.

**Methods:**

Immunohistochemistry was used to detect autophagy marker LC3B in human and murine diabetic and non-diabetic retinas. Cultured human retinal capillary pericytes (HRCPs) were treated with in vitro-modified heavily-oxidised glycated LDL (HOG-LDL) vs native LDL (N-LDL) with or without autophagy modulators: green fluorescent protein–LC3 transfection; small interfering RNAs against Beclin-1, c-Jun NH(2)-terminal kinase (JNK) and C/EBP-homologous protein (CHOP); autophagy inhibitor 3-MA (5 mmol/l) and/or caspase inhibitor Z-VAD-fmk (100 μmol/l). Autophagy, cell viability, oxidative stress, endoplasmic reticulum stress, JNK activation, apoptosis and CHOP expression were assessed by western blots, CCK-8 assay and TUNEL assay. Finally, HOG-LDL vs N-LDL were injected intravitreally to STZ-induced diabetic vs control rats (yielding 50 and 200 mg protein/l intravitreal concentration) and, after 7 days, retinas were analysed for ER stress, autophagy and apoptosis.

**Results:**

Intra-retinal autophagy (LC3B staining) was increased in diabetic vs non-diabetic humans and mice. In HRCPs, 50 mg/l HOG-LDL elicited autophagy without altering cell viability, and inhibition of autophagy decreased survival. At 100–200 mg/l, HOG-LDL caused significant cell death, and inhibition of either autophagy or apoptosis improved survival. Further, 25–200 mg/l HOG-LDL dose-dependently induced oxidative and ER stress. JNK activation was implicated in autophagy but not in apoptosis. In diabetic rat retina, 50 mg/l intravitreal HOG-LDL elicited autophagy and ER stress but not apoptosis; 200 mg/l elicited greater ER stress and apoptosis.

**Conclusions:**

Autophagy has a dual role in diabetic retinopathy: under mild stress (50 mg/l HOG-LDL) it is protective; under more severe stress (200 mg/l HOG-LDL) it promotes cell death.

**Electronic supplementary material:**

The online version of this article (doi:10.1007/s00125-016-4058-5) contains peer-reviewed but unedited supplementary material, which is available to authorised users.

## Introduction

Diabetic retinopathy remains a major cause of visual impairment in the working-age population [1]. Pericytes are critical in maintaining retinal vascular integrity [2]; their loss is considered an initiating event of diabetic retinopathy [3–6] but the mechanisms of pericyte loss are poorly understood, hindering development of effective therapies.

Autophagy, a catabolic process by which cells degrade and recycle their own constituents through a lysosomal mechanism, acts as a cellular sensor of intra- and extracellular stresses [7, 8]. In diabetes, misfolded proteins accumulate in the endoplasmic reticulum (ER) leading to ER stress. ER stress activates the unfolded protein response (UPR), restoring protein homeostasis and promoting cell survival. Autophagy is an alternative pathway to the UPR. Rapamycin, an inducer of autophagy, inhibits angiogenic sprouting and vascular endothelial growth factor (VEGF) production in a co-culture model of retinal pigment epithelial (RPE) and endothelial cells [9–11], and in diabetic rats it suppresses retinal oxidative stress and VEGF expression [10] and prevents age-related retinopathy [11]. Targeting autophagy may therefore have therapeutic potential; however, in certain circumstances, autophagy may activate apoptotic death [12], and depending on context, stress-induced autophagy may promote survival or death of a given cell species [13].

Diabetic retinopathy is generally viewed as a consequence of hyperglycaemia, but in previous studies we showed that extravasated modified LDL also plays a critical role [14–24]. The effects of extravasated lipoproteins in atherosclerosis are well-established [25]; we have demonstrated analogous effects in the retina once the blood–retinal barrier (BRB) integrity is compromised, or bypassed, as in an animal model we described recently [26]. Initial leakage may be mild and transient but, as extravasated LDL accumulates, vicious cycles of damage may be established. While oxidised lipoprotein-induced autophagy is implicated in atherogenesis [27], little is known regarding the retina. Previously, we showed that in human retinal capillary pericytes (HRCPs) and retinal pigment epithelium (RPE), ER stress that had been induced by highly oxidised glycated human LDL is mitigated by the UPR, but that in the presence of continued severe stresses ER homeostasis could not be preserved, resulting in apoptosis [22, 23]. In this study, we aim to determine the underlying mechanisms whereby autophagy and apoptosis contribute to pericyte death.

## Methods

### Ethics

The study was approved by the Institutional Review Board at the University of Oklahoma Health Sciences Centre (OUHSC) and the Ethics Committee at the Queen’s University of Belfast, and was conducted according to the principles of the Declaration of Helsinki. Animal experiments were approved by the Institutional Animal Care and Use Committee at the Medical University of South Carolina and by the Queen’s University Belfast Ethical Review Committee for Animal Research. All the animal experiments were randomised and blind to group assignment and outcome assessment.

### Immunohistochemistry of human retinas

Human retinas were obtained post-mortem from the National Disease Research Interchange (NDRI; Philadelphia, PA, USA) as described [23, 24]. Retinas were from age-matched individuals categorised as follows: non-diabetic, diabetic without clinical diabetic retinopathy; diabetic with retinopathy (*n* = 3 or 4 per group). The antibody used for immuno-histochemical detection of autophagy marker LC3B (catalogue No. 3868, 1:100 dilution; Cell Signaling Technology, Danvers, MA, USA) was also used in western blots (below): according to the manufacturer, it recognises human and murine LC3BI and LC3BII, and may exhibit some cross-reactivity with LC3A. Absence of non-specific tissue binding by secondary antibodies was confirmed. See Methods in electronic supplementary material (ESM) for details of retinal sample preparation and immunohistochemistry.

### LDL preparation, modification and characterisation

Lipoproteins were prepared as described [23, 28]. Briefly, native LDL (N-LDL) was prepared by sequential ultracentrifugation of freshly pooled plasma from healthy human volunteers. Highly oxidised glycated LDL (HOG-LDL) was prepared by glycating N-LDL, then oxidising with CuCl_2_. See ESM Methods for details.

### Genetically modified mouse model of hyperlipidaemia

Genetically modified C57B16 male mice (Genentech, South San Francisco, CA, USA) with double knockout of the genes encoding the LDL receptor (*Ldlr*^−/−^) and apolipoprotein B mRNA-editing catalytic polypeptide (converts ApoB100 to ApoB48) (*Apobec1*^−/−^) were used to model hypercholesterolaemia (vs wild-type [WT] controls) [29]. When mice reached 7 weeks of age, diabetes was induced by streptozotocin (STZ) as described [30], yielding groups with and without diabetes and with and without hypercholesterolaemia. All mice were maintained under a 12 h light–12 h dark cycle (07:00–19:00 hours) and constant temperature (25°C) throughout the study, with food and water given ad libitum. Retinas were studied 40 weeks after diabetes induction: see ESM Methods for details.

### Diabetes induction and LDL intravitreal injection in rats

Diabetes was induced by STZ in adult (8–10 weeks) male Sprague–Dawley rats weighing 280–330 g. After 8 weeks, human HOG-LDL or N-LDL (5 μl, 0.5 or 2.0 g protein/l in PBS, to yield 50 or 200 mg/l final intravitreal concentration), or PBS alone, was injected intravitreally. After 7 days, retinas were harvested for western blot. The rats were maintained under a 12 h light–12 h dark cycle (07:00–19:00 hours) and constant temperature (25°C) throughout the study, with food and water given ad libitum. See ESM Methods for details.

### HRCP cell culture

HRCPs (Cambrex, Walkersville, MD, USA) were cultured in EBM-2 medium. Cells (passages 3–9) at 85% confluence were treated with either N-LDL or HOG-LDL as indicated. Where appropriate, cells were pre-treated with pharmacological reagents prior to lipoprotein exposure. See ESM Methods for details.

### Cell viability assay

HRCPs were studied in 96-well plates (1 × 10^4^ cells/well). Cell viability was measured using a cell counting assay (CCK-8; Dojindo Molecular Technologies, Rockville, MD, USA), per the manufacturer’s protocol.

### Western blotting

Human retinas or pericytes were homogenised with a complete lysis buffer (Roche, Indianapolis, IN, USA). Protein concentrations were determined by the BCA protein assay (Pierce, Rockford, IL, USA). Protein (30 μg) was resolved by SDS-PAGE and then blotted with antibodies against autophagy-related homologue 5 (ATG-5), Beclin-1, LC3B (detects both LC3B-I and LC3B-II; some cross-reactivity with LC3A), phosphorylated c-Jun NH(2)-terminal kinase (p-JNK), total JNK, cleaved poly ADP ribose polymerase (PARP), activated caspase-3, C/EBP-homologous protein (CHOP), β-actin (1:3000) (all from Cell Signaling Technology); and phosphorylated‑protein kinase R‑like ER kinase (p‑PERK), 78 kDa glucose-regulated protein (GRP78) and phosphorylated eukaryotic initiation factor 2α (eIF2α) (all from Abcam, Cambridge, MA, USA). All antibodies were diluted 1:1000 unless indicated otherwise. Antibody to β-actin was used as a loading control. All cell culture experiments were repeated three times independently and quantified by densitometry.

### TUNEL assay for apoptosis

HRCPs were seeded and grown to 85% confluence on glass coverslips, and treated as indicated. Apoptosis was assessed (in situ cell death detection kit; Roche, Indianapolis, IN, USA) as per manufacturer’s instructions. Immunofluorescence was visualised under a fluorescence microscope (Nikon, Tokyo, Japan).

### Measurement of intracellular reactive oxygen species

Reactive oxygen species (ROS) were measured with chloromethyl derivative of H*2*DCFDA (CM-H*2*DCFDA) (Life Technologies, Invitrogen, Carlsbad, CA, USA) as previously described [24]. Briefly, cells were seeded in 96-well plates (1 × 10^4^ cells/well). When they reached 80% confluence, cells were washed and incubated with 20 μmol/l DCFDA at 37°C for 20 min, then exposed to experimental conditions. Fluorescence was measured at an excitation wavelength of 495 nm and an emission wavelength of 525 nm (VICTOR3 microplate reader; PerkinElmer, Waltham, MA, USA).

### Data analyses

Data are expressed as means ± SD. Statistical significance was determined by Student’s *t* test or one-way ANOVA followed by post hoc Dunnett’s test as appropriate (Prism 5 software; Graphpad, La Jolla, CA, USA). A *p* value of ≤ 0.05 was considered significant.

## Results

### Autophagy in human diabetic retina

**Fig. 1:**
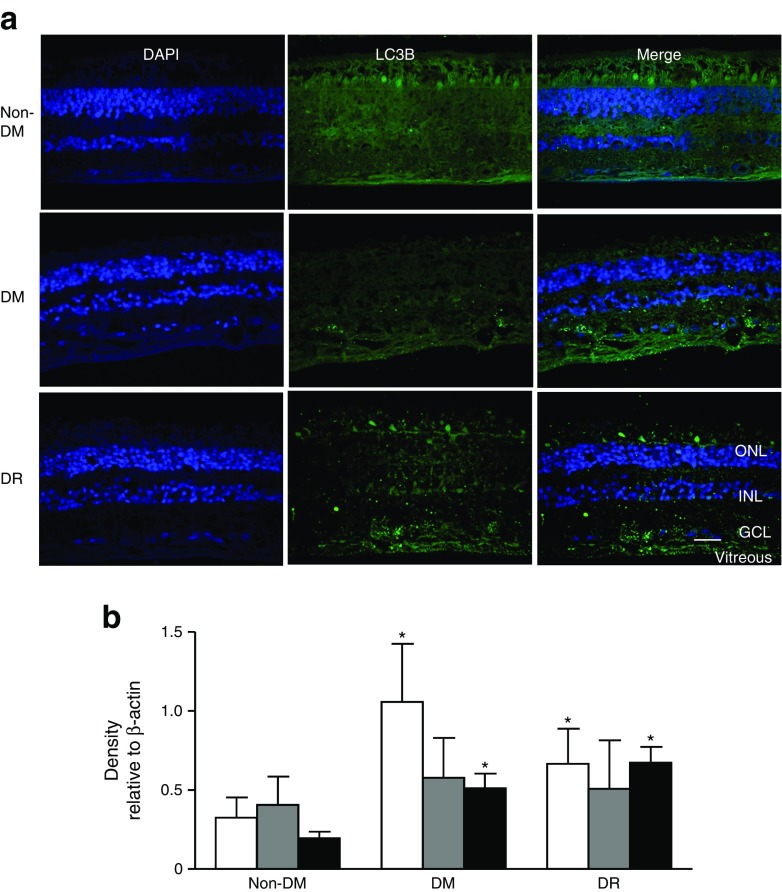
Autophagy is evident in diabetic human retinas. (**a**) Immunohistochemistry for LC3B in human retinal sections: non-diabetic (non-DM), diabetic without clinical retinopathy (DM) and diabetic with clinical retinopathy (DR). DAPI (blue) was used to visualise the nuclei. Scale bar, 20 μm. GCL, ganglion cell layer; INL, inner nuclear layer; ONL, outer nuclear layer. Punctate staining of LC3B (green) was present in both groups of diabetic retinas but was minimal in non-diabetic retinas. There was no obvious difference between the two diabetic groups. (**b**) Western blots for ATG-5 (white bars), Beclin-1 (grey bars) and LC3B (black bars) were performed on total retinal protein extracts from individual human retinas and quantified by densitometry (mean ± SD, *n* = 3 or 4, *p < 0.05 vs non-DM)

LC3B immunohistochemistry was performed in retinas from individuals with type 2 diabetes with and without diabetic retinopathy, and from non-diabetic individuals. In diabetic retinas, punctate staining (indicating autophagosomes) was observed in the ganglion cell layer and inner nuclear layer, but in non-diabetic retinas, punctate staining was absent (Fig. 1a). Retinal protein lysates were analysed (western blotting) for LC3B and two other autophagy markers, ATG-5 and Beclin-1. LC3B and ATG-5 were higher in diabetic vs non-diabetic individuals, but retinopathy status had no effect; Beclin-1 levels tended to be higher in diabetic retinas (Fig. 1b). Overall, autophagy was increased in the diabetic retina; the similarity between those with and without retinopathy may reflect pre-clinical injury in people who appear disease-free.

### Autophagy in diabetic and hypercholesterolaemic mouse retina

LC3B staining was significantly higher in the two diabetic groups vs the non-diabetic group, with hyperlipidaemic diabetic mice showing the greatest intensity, localised predominantly in the ganglion cell and inner nuclear layers (ESM Fig. 1). Again, retinal autophagy was increased in the presence of diabetes, and more so in the added presence of long-standing hypercholesterolaemia.

### HOG-LDL induces autophagy in pericytes

**Fig. 2:**
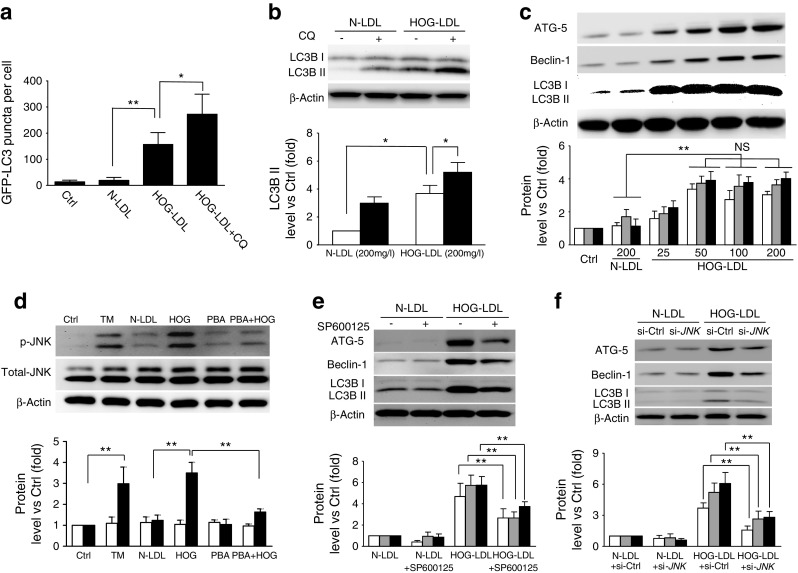
HOG-LDL induces JNK phosphorylation-dependent autophagy in HRCPs. (**a**) HRCPs were transfected with GFP–LC3B plasmid for 36 h, then exposed to N-LDL (200 mg/l) or HOG-LDL (200 mg/l) for 24 h, with or without CQ (10 μmol/l) pre-treatment for 1 h. Untreated cells served as control (Ctrl) in this and the following figs. Autophagy was quantified by counting GFP–LC3B puncta within cells (*n* = 5 experiments; *p < 0.05, **p < 0.01). (**b**) HRCPs were directly exposed to LDL with or without CQ and analysed by western blot for LC3BII. White bars, control; black bars, CQ (mean ± SD, *n* = 3; *p < 0.05). (**c**) HRCPs were exposed to N-LDL (200 mg/l) or HOG-LDL (25–200 mg/l) for 12 h and analysed by western blot. ATG-5 (white bars), Beclin-1 (grey bars) and LC3BII (black bars) levels were quantified (mean ± SD, *n* = 3; **p < 0.01). (**d**) HRCPs were treated with tunicamycin (TM; 2 μmol/l), N-LDL (200 mg/l) or HOG-LDL (200 mg/l) for 12 h or pre-treated with 4-phenylbutyric acid (PBA; 10 mg/l) before exposure to HOG-LDL. Western blot analysis was carried out and total JNK (white bars) and p-JNK (black bars) levels were quantified (mean ± SD, *n* = 3; **p < 0.01). (**e**, **f**) HRCPs were pre-treated with p-JNK inhibitor SP600125 (10 μmol/l) for 1 h (**e**), or transfected with siRNA against JNK (si-*JNK*) or with si-Ctrl for 36 h (**f**), then exposed to N-LDL (200 mg/l) or HOG-LDL (200 mg/l) for 12 h. Western blotting was carried out and levels of ATG-5 (white bars), Beclin-1 (grey bars) and LC3BII (black bars) were quantified (mean ± SD, *n* = 3; **p < 0.01)

To determine whether modified LDL elicits autophagy in vitro, cultured HRCPs were transfected with a green fluorescent protein (GFP)-labelled LC3 plasmid, then exposed to HOG-LDL vs N-LDL. HOG-LDL elicited a punctate intracellular GFP–LC3B distribution, characteristic of autophagy [31], which was not observed in response to N-LDL (Fig. 2a and ESM Fig. 2). The effect was further enhanced by chloroquine (CQ), an autophagosome–lysosome fusion blocker [32], confirming that HOG-LDL enhances autophagic flux. In concert, western blots of HRCP lysates showed increased levels of LC3BII (the lipidated form present in autophagosomes) in response to HOG-LDL vs N-LDL, and a further increase in response to HOG-LDL + CQ (Fig. 2b). HOG-LDL increased protein expression of ATG-5, Beclin-1 and LC3BII in a dose-dependent manner over the concentration range 0–50 mg/l, but caused no further increase at concentrations from 50 to 200 mg/L (Fig. 2c). Concentrations > 200 mg/l were not tested due to cellular toxicity.

### JNK mediates HOG-LDL-induced ER stress and autophagy

Jun amino-terminal kinases have been implicated in stress-induced autophagy [33, 34]. In cultured HRCPs, HOG-LDL vs N-LDL significantly increased JNK phosphorylation (Fig. 2d), to an extent equivalent to that induced by tunicamycin, an ER stress inducer. This response was obliterated by pre-treatment with sodium phenylbutyrate, an ER stress inhibitor, confirming that JNK mediates HOG-LDL-induced ER stress in pericytes.

To determine the role of JNK in autophagy, JNK phosphorylation inhibitor SP60012 was employed. JNK pathway inhibition was confirmed by western blot (ESM Fig. 3a). SP60012 pre-treatment attenuated HOG-LDL-induced autophagy, as demonstrated by decreased expression of LC3BII, ATG-5 and Beclin-1 (Fig. 2e). JNK knockdown using small interfering RNA (siRNA) had a similar effect, reducing both phosphorylated and total JNK (ESM Fig. 3b) as well as HOG-LDL-induced autophagy (Fig. 2f). The data support an essential role for JNK activation in the mediation of HOG-LDL-induced autophagy in HRCPs.

### A dual role for autophagy in HOG-LDL-induced pericyte death

**Fig. 3:**
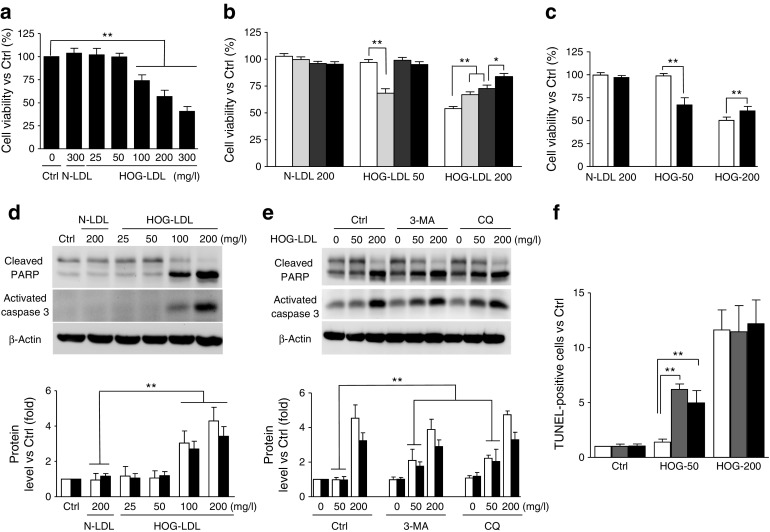
A dual role for autophagy in HOG-LDL-induced pericyte death. (**a**–**c**) HRCPs were treated as follows: exposure to N-LDL (300 mg/l) or HOG-LDL (25–300 mg/l) for 24 h (**a**); pre-treatment with 3-MA (5 mmol/l, light grey bars), Z-VAD-fmk (100 μmol/l, dark grey bars) and both of them (black bars) for 1 h (**b**); transfection with siRNA against Beclin-1 (si-*Beclin*-1, black bars) or si-Ctrl (white bars) for 36 h, then exposure to HOG-LDL (50 mg/l, 200 mg/l) for 12 h (**c**). Cell viability was expressed as percentage vs control. (**d-f**) HRCPs: apoptosis and autophagy. Apoptosis is triggered by HOG-LDL at 100 or 200 mg/l: cleaved PARP (white bars), activated caspase-3 (black bars) were detected by western blot (**d**). HRCPs were pre-treated with 3-MA (5 mmol/l) or CQ (10 μmol/l) for 1 h: cleaved PARP (white bars) and activated caspase-3 (black bars) were detected by western blot (**e**). HRCPs were pre-treated with vehicle (white bars) or 3-MA (5 mmol/l, grey bars) for 1 h, or were transfected with si-*Beclin*-1 (black bars), then exposed to HOG-LDL (50 mg/l, 200 mg/l) for 12 h: TUNEL staining was expressed as a ratio of control (**f**). All data are means ± SD, *n* = 3 or 5; *p < 0.05, **p < 0.01. Ctrl, control

HOG-LDL caused dose-dependent toxicity to cultured HRCPs: no death occurred up to 50 mg/l, but viability decreased progressively from 50 mg/l to 300 mg/l (Fig. 3a). To understand the relative involvement of autophagy and apoptosis, we employed 3-methyladenine (3-MA), a specific inhibitor of phosphoinositide 3-kinase and the initial phase of autophagy, and Z-VAD-fmk, a caspase inhibitor. As shown in Fig. 3b, at a non-toxic HOG-LDL concentration of 50 mg/l, 3-MA triggered pericyte death, which was prevented by the apoptosis inhibitor Z-VAD. This suggests a pro-survival role for autophagy under mild, sub-lethal stress (50 mg/l HOG-LDL). However, at a higher HOG-LDL concentration (200 mg/l), 3-MA attenuated cell death, adding to the rescuing effect of Z-VAD (Fig. 3b). As 3-MA may not provide specific inhibition of autophagy, siRNA against Beclin-1 was also used: Beclin-1 knockdown decreased pericyte viability after exposure to 50 mg/l HOG-LDL but enhanced it after exposure to 200 mg/l HOG-LDL (Fig. 3c). The data suggest that under severe cellular stresses, autophagy shifts from a protective to an injurious role. This observation was further supported by western blotting analysis and TUNEL assay (Fig. 3d–f). HOG-LDL induced apoptosis at concentrations of 100 and 200 mg/l, demonstrated by increased levels of cleaved PARP, activated caspase 3 and TUNEL-positive apoptotic cells at 200 mg/l but not at 50 mg/l. However, inhibition of autophagy by 3-MA and CQ induced apoptosis at a HOG-LDL concentration of 50 mg/l but had no effect at 200 mg/l, indicating the protective role of autophagy at mild but not at severe levels of HOG-LDL-induced stress (Fig. 3d–f and ESM Fig. 4a).

### Comparison of the dose–response relationships for HOG-LDL-induced oxidative stress, ER stress, JNK activation and CHOP expression

**Fig. 4:**
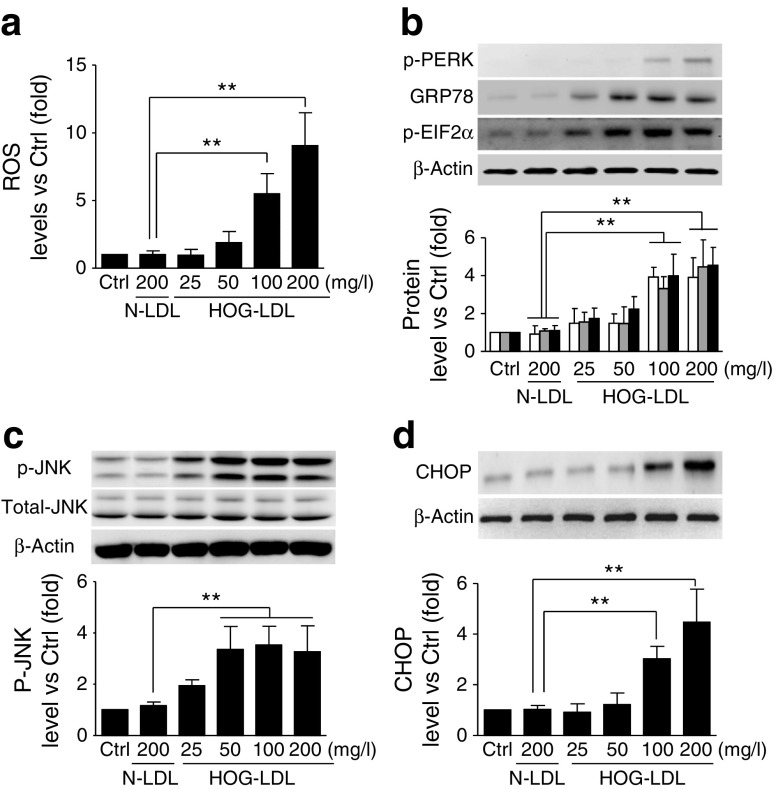
HOG-LDL dose–responses for ROS generation, ER stress, JNK activation and CHOP expression. Cellular ROS level are shown following exposure to various doses of HOG-LDL (mean ± SD, *n* = 3; **p < 0.01 vs N-LDL) (**a**). Western blots are shown for p-PERK (white bars), GRP78 (grey bars) and p-eIF2α (black bars) (**b**), p-JNK and total JNK (**c**), and CHOP (**d**). Data are means ± SD, *n* = 3; **p < 0.01. Ctrl, control

To elucidate underlying signalling pathways and mechanisms, we compared dose–response relationships of oxidative stress (ROS), ER stress (chaperone: GRP78; sensors: p-PERK, p-eIF2α), JNK activation and CHOP expression in cultured HRCPs exposed to HOG-LDL. HOG-LDL increased levels of ROS, GRP78, p-PERK and p-eIF2α dose-dependently (25–200 mg/l) (Fig. 4a, b). JNK activation increased over the 25–50 mg/l HOG-LDL range, then remained constant up to 200 mg/l (Fig. 4c), similar to autophagy (Fig. 2c). HOG-LDL did not increase CHOP expression until its concentration reached 100 mg/l (Fig. 4d), consistent with effects on apoptosis (Fig. 3d). Together, the data suggest that low-dose HOG-LDL (up to 50 mg/ml) induces mild oxidative and ER stress, triggering a protective action of autophagy via JNK; at higher concentrations (100–200 mg/l), HOG-LDL further increases stresses leading to autophagic and apoptotic death.

### CHOP, not JNK, is responsible for HOG-LDL-induced apoptosis

**Fig. 5:**
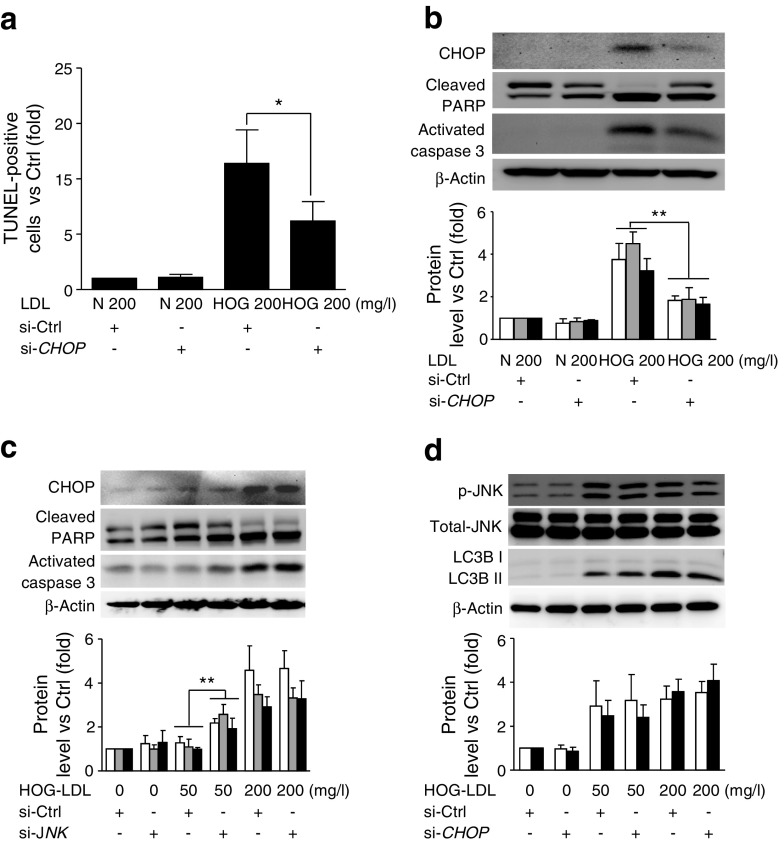
CHOP, but not JNK, is essential for HOG-LDL-induced apoptosis. HRCPs were transfected with si-*CHOP* or si-*JNK* for 36 h, then exposed to N-LDL (N; 200 mg/l) or HOG-LDL (HOG; 50, 200 mg/l) for 12 h. At 200 mg/l HOG-LDL, si-*CHOP* decreased TUNEL-positive cells (**a**). At 200 mg/l, si-*CHOP* decreased HOG-LDL-induced expression of CHOP (white bars), cleaved PARP (grey bars) and activated caspase-3 (black bars) (**b**). At 50 mg/l HOG-LDL, si-*JNK* increased expression of CHOP (white bars), cleaved PARP (grey bars) and activated caspase-3 (black bars) but had no effect at 200 mg/l (**c**). si-*CHOP* did not alter expression of p-JNK (white bars) or LC3BII (black bars) at 50 or 200 mg/l HOG-LDL (**d**). Data are means ± SD, *n* = 3; *p < 0.05, **p < 0.01

Both CHOP and JNK have been implicated in ER stress-induced apoptosis [35]. To determine their relative roles in HOG-LDL-induced apoptosis, we employed siRNA against *CHOP* (si-*CHOP*) or *JNK* (si-*JNK*), then measured apoptosis (TUNEL assay, western blots). si-*CHOP* significantly reduced TUNEL-positive staining (Fig. 5a, ESM Fig. 4b), cleaved PARP and activated caspase-3 (Fig. 5b), indicating a role for CHOP in apoptosis. However, in pericytes exposed to 50 mg/l HOG-LDL, si-*JNK* increased protein levels of CHOP, cleaved PARP and activated caspase-3 (Fig. 5c), responses that promote apoptosis. In contrast, in the presence of 200 mg/l HOG-LDL, si-*JNK* had no effect. This is consistent with JNK knockdown inhibiting autophagy, thus blocking the protective effects of autophagy at lower levels of cell stress. Finally, we showed that si-*CHOP* did not change expression of p-JNK or LC3BII in pericytes exposed to HOG-LDL at 50 or 200 mg/l (Fig. 5d), indicating that autophagy induced by HOG-LDL, in contrast to apoptosis, was CHOP-independent.

### Extravascular HOG-LDL dose-dependently induced ER stress, autophagy and apoptosis in diabetic rat retinas

**Fig. 6:**
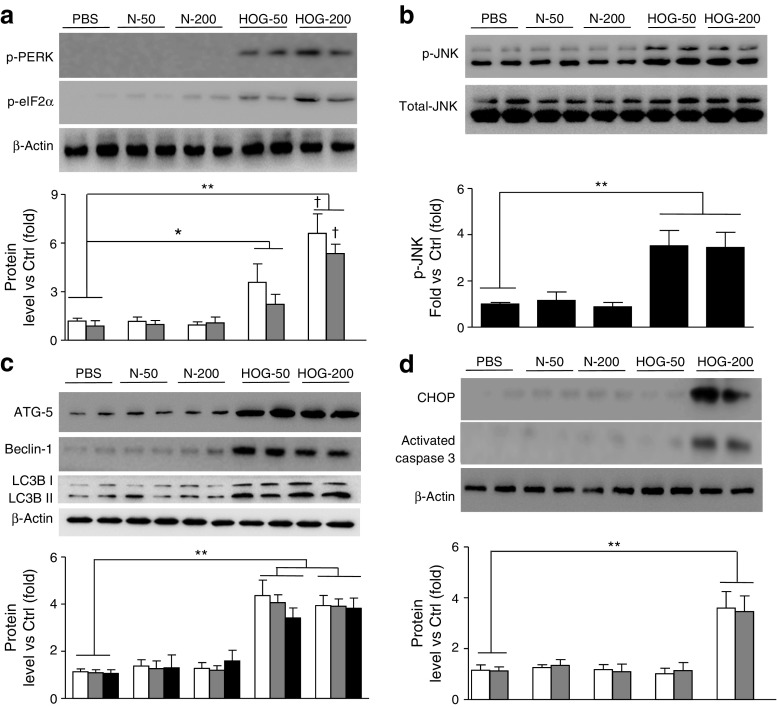
Effects of intravitreal HOG-LDL on ER stress, JNK activation, autophagy and apoptosis in retinas of diabetic rats. STZ-induced diabetic rats received intravitreal HOG-LDL (HOG) vs N-LDL (N) to achieve vitreal levels of 50 or 200 mg protein/l vs PBS control. Total retinal protein extracts were analysed by western blot, and the following protein levels were quantified by densitometry: p-PERK (white bars) and p-eIF2α (grey bars) (**a**); p-JNK and total JNK (**b**); ATG-5 (white bars), Beclin-1 (grey bars) and LC3BII (black bars) (**c**); CHOP (white bars) and activated caspase 3 (grey bars) (**d**). Data are means ± SD, *n* = 5; *p < 0.05 and **p < 0.01 vs PBS; ^†^ *p* < 0.05 vs HOG-50

To define responses in vivo, we evaluated retinas of diabetic rats, in which human N-LDL or HOG-LDL had been injected 7 days previously into the vitreous, to simulate chronic exposure to extravasated, modified LDL in human diabetic retinopathy, as recently described in a mouse model [26]. Two intravitreal concentrations, 50 and 200 mg/l, were used to induce different degrees of retinal stress. Intravitreal HOG-LDL, but not N-LDL, induced ER stress, autophagy and apoptosis (Fig. 6). ER stress sensors (p-PERK, p-eIF2a) increased in a dose-dependent manner (Fig. 6a), whereas p-JNK and autophagy markers were increased to a similar extent at both doses (Fig. 6b, c). Apoptosis (CHOP, activated caspase 3) was observed only at 200 mg/l HOG-LDL (Fig. 6d). These findings are in agreement with the cell culture studies.

## Discussion

**Fig. 7:**
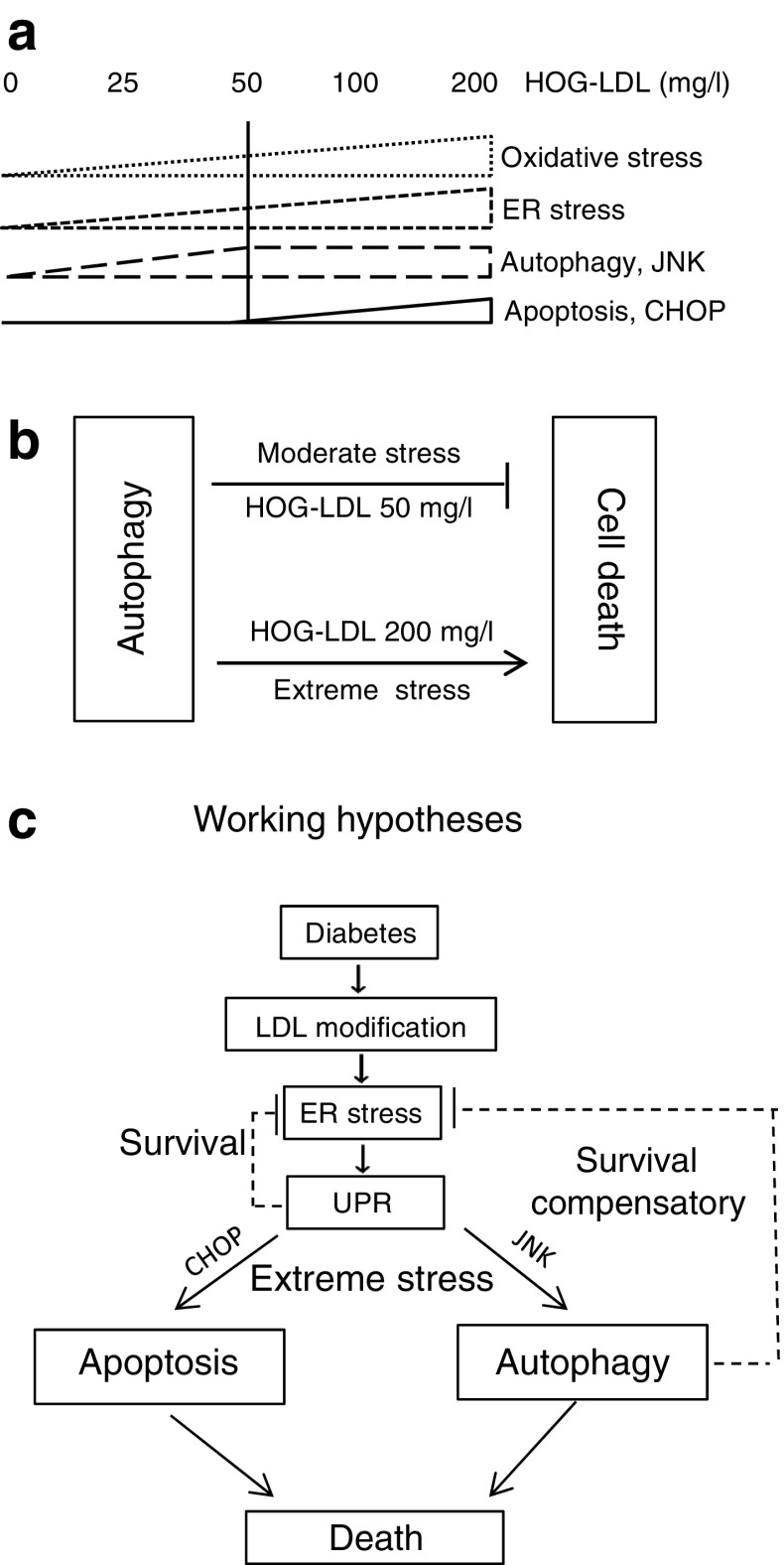
Dual role for autophagy in stress-induced pericyte loss in diabetic retinopathy. (**a**) Summary of dose-dependent effects of HOG-LDL on oxidative stress, ER stress, autophagy and apoptosis in HRCPs. (**b**) Contrasting roles of autophagy in moderate vs severe HOG-LDL-induced cell stress. (**c**) Study hypothesis: in diabetic retina, modified LDL-triggered ER stress may be overcome by UPR or autophagy (as a pro-survival, compensatory pathway) but if cellular stress becomes more severe, both UPR and autophagy may shift to promote apoptotic and autophagic cell death

Therapies targeting autophagy are of increasing interest and may be effective in retinal disease [9–11] but little is known regarding diabetic retinopathy. In the current study, we showed for the first time that autophagy markers (by immunohistochemistry and western blot) were increased in human retinas in the presence of diabetes, with or without concomitant retinopathy, and that in cell culture, exposure of pericytes to modified lipoproteins stimulated autophagy. These findings are consistent with our overall hypothesis that ectopic (extravasated) modified lipoproteins, when present in the diabetic retina, mediate responses (some defensive, some injurious) even before clinical diabetic retinopathy is evident [21, 23]. More interesting, our in vitro and in vivo data both suggest that autophagy may play a dual role: protecting against cell death under moderate stress, but contributing to it under severe stress (Fig. 7).

To maintain normal cellular function, autophagy may be upregulated in response to environmental stress. In the retina, all cell types rely on one or more aspects of autophagy to maintain structure and/or function [36]. Retinal autophagy was first observed (in rats) by Remé et al in 1977 [37], occurring primarily in the outer retina, where later it was shown to exhibit circadian variation [38]. More recently, Piano et al demonstrated upregulation of autophagy in retinal rods as an early feature of diabetic retinopathy (i.e. after 4–12 weeks diabetes) in STZ-induced diabetic mice [39]. In general, cellular homeostasis relies on regulated interplay between basal and stress-induced autophagic pathways [40]. Previously we showed that both extravasated modified lipoproteins and markers of ER stress were present in human diabetic retinas in proportion to the severity of retinopathy [23]. In the present study, the retinal findings from humans and genetically hypercholesterolaemic mice, with and without diabetes, are generally consistent with the findings described above: intra-retinal formation of autophagosomes (punctate LC3 staining) was increased by diabetes and further increased (in mice) by prolonged hypercholesterolaemia. In a new diabetic rat model using intravitreal LDL injection, we found additional supportive evidence: exposure of the retina to HOG-LDL but not N-LDL stimulated an autophagic response. Taken together, the data are consistent with autophagy being implicated in lipoprotein-mediated retinal injury and, specifically, from the cell culture work, in pericyte injury in diabetic retinopathy.

From the present data, we propose that the interplay between autophagy and apoptosis is critical for pericyte survival. The balance between survival and death depends on the level of stress—minor stress may be countered by autophagy but severe stress leads to cell death. This concept is in concert with the findings of Piano et al regarding effects of early diabetic retinopathy on retinal neural cells [39]. In the present work, the survival–death balance is seen in the observed dose-dependent effects of HOG-LDL on pericyte oxidative stress, ER stress, apoptosis and autophagy, with consistent findings following intravitreal LDL injections in rats, summarised in Fig. 7. HOG-LDL induced various molecular responses that were dose-dependent over different concentration ranges. Oxidative stress and ER stress were dose-dependent up to 200 mg/l, autophagy up to 50 mg/l and apoptosis from 50 mg/l to 200 mg/l. When cells experienced relatively mild stress (<50 mg/l), autophagy was induced, promoting cell survival, but under more severe stress (100 or 200 mg/l), autophagy was no longer protective but instead contributed to disruption of cellular homeostasis and death. Thus, pericytes utilise autophagy as a cytoprotective mechanism unless, or until, a critical stress threshold is exceeded. At that point, neither the UPR nor autophagy can protect the cells; instead, apoptotic mechanisms are activated and autophagy shifts from a protective to a lethal role. The definition of 50 mg/l as the threshold for this shift is clearly inexact, but nevertheless relevant in the retina where LDL is normally excluded by the BRB: any degree of BRB leakage would likely lead to accumulation and transition through this value. The pathophysiological relevance of the concentrations of HOG-LDL (0–200 mg/l) used in this study has been described and justified previously [21, 23], and we believe them to be relevant in vivo.

A dual role for autophagy has been proposed in other studies. In rheumatoid arthritis, autophagy in synovial fibroblasts may promote cell survival or death, depending on the level of stress [41]. In the liver, autophagy is essential for maintenance of hepatocyte mitochondria and control of oxidative stress, thus preventing carcinogenesis, but once hepatocarcinoma is established, autophagy can promote the disease [42]. In diabetic retinopathy, apparently contradictory roles for autophagy can inhibit or promote retinal vascular injury, depending on context (e.g. severity of retinopathy, extent of LDL leakage, extent of modification). In early retinopathy, when BRB leakage is mild, the retina is exposed only to small quantities of modified LDL [18] and autophagy may enhance cell survival. As retinopathy progresses and BRB leakage and lipoprotein leakage/modification become more severe, neither UPR nor autophagy can maintain ER homeostasis and autophagic death ensues. This dual action may complicate the development of treatments for diabetic retinopathy that aim to modulate autophagy.

The JNK pathway has been implicated in a range of cellular stress responses [7, 33, 43]. In this study, we demonstrated that JNK phosphorylation was essential to autophagy induced by HOG-LDL and ER stress. This is consistent with data implicating PERK–eIF2a and IRE1–JNK signalling pathways in autophagy [7, 44, 45]. Apart from autophagy, JNK activation is implicated in ER stress-induced apoptosis [46]. However, in the present study, we showed that JNK ‘knockdown’ did not affect HOG-LDL (200 mg/l)-induced apoptosis, and enabled lower concentrations of HOG-LDL (50 mg/l) to induce apoptosis. This suggests that JNK activation was not implicated in HOG-LDL-induced apoptosis, but at low levels of stress it may promote autophagy and thus protect cells against apoptosis. It remains unclear whether or how activation of JNK through ER stress affects upstream components of the autophagy pathway (e.g. mechanistic target of rapamycin; mTOR) [47]. Further studies regarding the detailed pathway of HOG-LDL-induced autophagy, including the relative roles of LC3B and LC3A, which may not be clearly distinguished in the present study, are needed: possibly, differential effects of the two isoforms could be important.

In conclusion, we present further evidence that autophagy is present in human diabetic retinas, and a role for modified lipoproteins is supported by in vivo findings in diabetic mouse and rat retina and by in vitro *s*tudies of HRCPs. We show that ER stress-mediated autophagy may play a dual role in pericyte loss induced by modified LDL. At low levels of exposure autophagy has a pro-survival effect, but as stresses become severe it promotes cell death. This dual function has implications for the development of any future autophagy-based therapies, which might only be applicable early in disease evolution when intra-retinal stresses remain mild.

## Electronic supplementary material

Below is the link to the electronic supplementary material.

ESM (PDF 586 kb)

## Abbreviations

3-MA: 3-Methyladenine
ATG-5: Autophagy-related homologue 5
BRB: Blood–retinal barrier
CHOP: C/EBP-homologous protein
CQ: Chloroquine
eIF2α: Eukaryotic initiation factor 2α
ER: Endoplasmic reticulum
GFP: Green fluorescent protein
GRP78: Glucose-regulated protein
HOG-LDL: Highly oxidised glycated LDL
HRCP: Human retinal capillary pericyte
JNK: c-Jun NH(2)-terminal kinase
N-LDL: Native LDL
PARP: Poly ADP ribose polymerase
PERK: Protein kinase R‑like endoplasmic reticulum kinase
RPE: Retinal pigment epithelium
ROS: Reactive oxygen species
siRNA: Small interfering RNA
STZ: Streptozotocin
UPR: Unfolded protein response
VEGF: Vascular endothelial growth factor
WT: Wild-type
