# Supplementary material for: Survival or death: a dual role for autophagy in stress-induced pericyte loss in diabetic retinopathy
Source: Diabetologia. 2016 Jul 30;59(10):2251–61. doi: 10.1007/s00125-016-4058-5 (PMC5016562; doi:10.1007/s00125-016-4058-5)
Supplement: Supplementary file 1 — (PDF 586 kb) [file 125_2016_4058_MOESM1_ESM.pdf]

Electronic Supplementary Material

Methods

Immunohistochemistry of human retinas

Retinas were obtained post-mortem from type 2 diabetic or non-diabetic humans through the National Disease Research Interchange (NDRI; Philadelphia, PA) as described previously [1, 2]. Subjects were grouped as follows: non-diabetic, diabetic without clinical DR, and diabetic with DR (n=3 or 4 in each group), based on information provided by NDRI. Ages were similar across the groups. The eyes had been fixed in 10% neutral-buffered formalin within 12h of death. For immunohistochemistry, retinal sections (5 µm) were prepared and incubated overnight with rabbit polyclonal anti-microtubule-associated protein 1 light chain 3 (LC3)B antibody (1:100 dilution, Cell Signalling Technology, Danvers, MA; recognizes both LC3BI and LC3BII), followed by detection with fluorescence-conjugated anti-rabbit antibody (Life Technologies, Carlsbad, CA) and confocal microscopy (Olympus, Japan) as described [1]. Absence of non-specific tissue binding by secondary antibodies was confirmed. Per the manufacturer, the antibody may cross-react to some extent with LC3A, and like LC3B, some variants of LC3A may be cleaved, de-lipidated, and incorporated (as LC3A-II) into autophagosomes [3].

Genetically modified mouse model of hyperlipidaemia

To assess the relevance of modified lipoproteins in the context of diabetes, we used an animal model combining diabetes with hypercholesterolaemia. Male genetically modified C57B16 mice (Genentech, South San Francisco, CA) with double knockout of the LDL receptor (Ldlr−/−) and the apolipoprotein (Apo) B mRNA-editing catalytic polypeptide (which enables the conversion of ApoB100 to ApoB48) (Apobec1−/−) were used with appropriate wild-type controls [4]. These animals express high concentrations of ApoB100-containing LDL, and develop severe atherosclerosis even with a normal diet. At age 7 weeks, half of the animals of each group were rendered diabetic with streptozotocin as previously described [5]. All animals were maintained under a 12hr light/12hr dark cycle; 7am-7pm) and constant temperature (25°C) throughout the study, with food and water given ad libitum. Forty weeks after streptozotocin treatment, animals were sacrificed and the eyes were removed, fixed in formalin, sectioned, and then immuno-stained with rabbit polyclonal anti-LC3B antibody (1:100 dilution, Cell Signalling Technology, Danvers, MA, as above) to detect retinal autophagy. Absence of non-specific tissue binding by secondary antibodies was confirmed.

LDL preparation, modification and characterization

Preparation of HOG-LDL followed a previous protocol [1, 6]. Briefly, freshly pooled plasma was obtained from 4-6 healthy, normolipidaemic, non-diabetic volunteers aged 20-40 years who were taking neither prescribed medications nor antioxidant vitamins. Native LDL (N-LDL; d=1.019-1.063 g/mL) was prepared by sequential ultracentrifugation. To prepare 'highly oxidised, glycated LDL' (HOG-LDL), N-LDL was glycated in 50 mM glucose (72h, 37°C) under antioxidant conditions (1 mM DTPA, 270 μM EDTA, under nitrogen), dialyzed to remove glucose and antioxidants, and then further oxidised in 10 μM CuCl2 for 24h at 37°C. After extensive dialysis, LDL protein concentration was quantified by the BCA assay (Pierce, Rutherford, IL). N-LDL and HOG-LDL were characterized by electrophoresis and confirmed free of endotoxin using a Limulus Amoebocyte Lysate assay.

LDL intra-vitreal injected rat model

Animals were bred and maintained within the Biological Research Unit at Queen's University Belfast. Adult male SD rats (8-10 weeks) were maintained under a 12hr light/12hr dark cycle; 7am-7pm) and constant temperature (25°C) throughout the study, with food and water given ad libitum. To induce diabetes, streptozotocin (STZ, 60 mg/kg) was given i.p. once to rats weighing 280-330g. Control, non-diabetic animals were given i.p. sodium citrate buffer (pH 4.6). One week post-induction, diabetes was confirmed by the presence of non-fasting blood glucose >16.7 mmol/L.

Eight weeks after diabetes induction, rats were anesthetized by I.P. injection of pentobarbital (70mg/kg in 33.3mg/ml solution), and pupils were dilated (topical phenylephrine hydrochloride (2.5%) and atropine sulphate (1%)) for 10 min. Then, 5μl intra-vitreal human N-LDL or HOG-LDL to yield either 50 or 200mg/l (final intra-vitreal concentration) were administered posterior to the superior corneal limbus (33-gauge needle, 10 μl syringe (Hamilton, Davidson & Hardy (Laboratory Supplies) Ltd)). In each rat, the right eye was injected with HOG-LDL and the left with N-LDL. Some eyes were injected with PBS as controls. Seven days post-injection, the rats were sacrificed and retinas were harvested and prepared for western blots. Subjects were grouped as follows: PBS only; N-, or HOG-LDL 50 mg/l; N-, or HOG-LDL 200 mg/l (n=5 in each group).

Human retinal capillary pericyte (HRCP) culture

HRCP (Cambrex, Walkersville, MD) were cultured in EBM-2 medium (glucose 5.5 mM) supplemented with the EGM-2 SingleQuot kit (Lonza, Allendale, NJ). Cells (passages 3-9) at 85% confluence were treated with N- or HOG-LDL at various concentrations and times, non-treated cells served as controls in all experiments. In some experiments, cells were first transfected with a GFP-LC3 plasmid (Addgene, MA) or siRNA (si-Beclin-1, si-

CHOP, si-JNK or si-Control) for 36h using Lipofectamine 2000 (Invitrogen, Carlsbad, CA), followed by N- or HOG-LDL treatment. Where appropriate, cells were pre-treated with pharmacological reagents including JNK phosphorylation inhibitor SP600125, the autophagy inhibitors 3-methyladenine (3-MA) and chloroquine (CQ), the pan-caspase inhibitor Z-VAD-fmk, the ER stress inducer tunicamycin (TM) and the ER stress inhibitor 4-phenyl butyric acid (PBA) (Sigma-Aldrich, St. Louis, MO) for 1h before lipoprotein treatment .

[1] Fu D, Wu M, Zhang J, et al. (2012) Mechanisms of modified LDL-induced pericyte loss and retinal injury in diabetic retinopathy. Diabetologia 55: 3128-3140

[2] Fu D, Yu JY, Wu M, et al. (2014) Immune complex formation in human diabetic retina enhances toxicity of oxidized LDL towards retinal capillary pericytes. J Lipid Res 55: 860-869

[3] Bai H, Inoue J, Kawano T, Inazawa J (2012) A transcriptional variant of the LC3A gene is involved in autophagy and frequently inactivated in human cancers. Oncogene 31: 4397-4408

[4] Powell-Braxton L, Veniant M, Latvala RD, et al. (1998) A mouse model of human familial hypercholesterolemia: markedly elevated low density lipoprotein cholesterol levels and severe atherosclerosis on a low-fat chow diet. Nat Med 4: 934-938

[5] Hammad SM, Powell-Braxton L, Otvos JD, Eldridge L, Won W, Lyons TJ (2003) Lipoprotein subclass profiles of hyperlipidemic diabetic mice measured by nuclear magnetic resonance spectroscopy. Metabolism 52: 916-921

[6] Jenkins AJ, Velarde V, Klein RL, et al. (2000) Native and modified LDL activate extracellular signal-regulated kinases in mesangial cells. Diabetes 49: 2160-2169

ESM Fig. 1

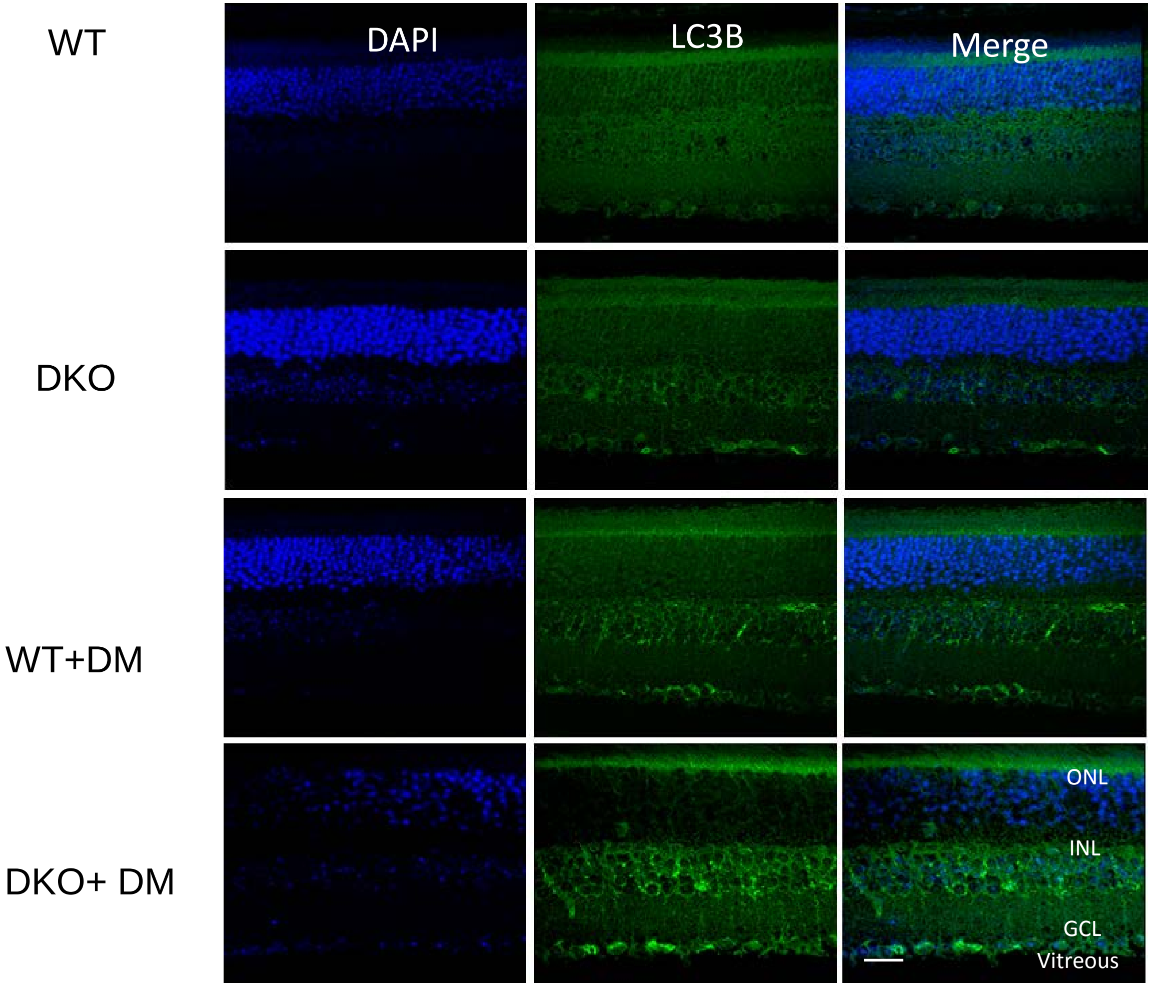

Autophagy is increased in retinas from diabetic and diabetic-hypercholesterolaemic STZ-diabetic mice. Immunohistochemistry for LC3B (green) was performed in mouse retinal sections wild-type (WT) and hyperlipidaemic (double knockout (DKO) for LDLR and Apobec1), each with and without STZ-induced diabetes (i.e. four groups). DAPI (blue) was used to visualize nuclei. Scale bar indicates 20 µm. ONL: outer nuclear layer; INL: inner nuclear layer; and GCL: ganglion cell layer. There was a clear increase of LC3B staining in the diabetic vs. the non-diabetic retinas in both mouse strains; and the intensity of staining was further increased in the presence of combined diabetes and hyperlipidaemia.

ESM Fig. 2

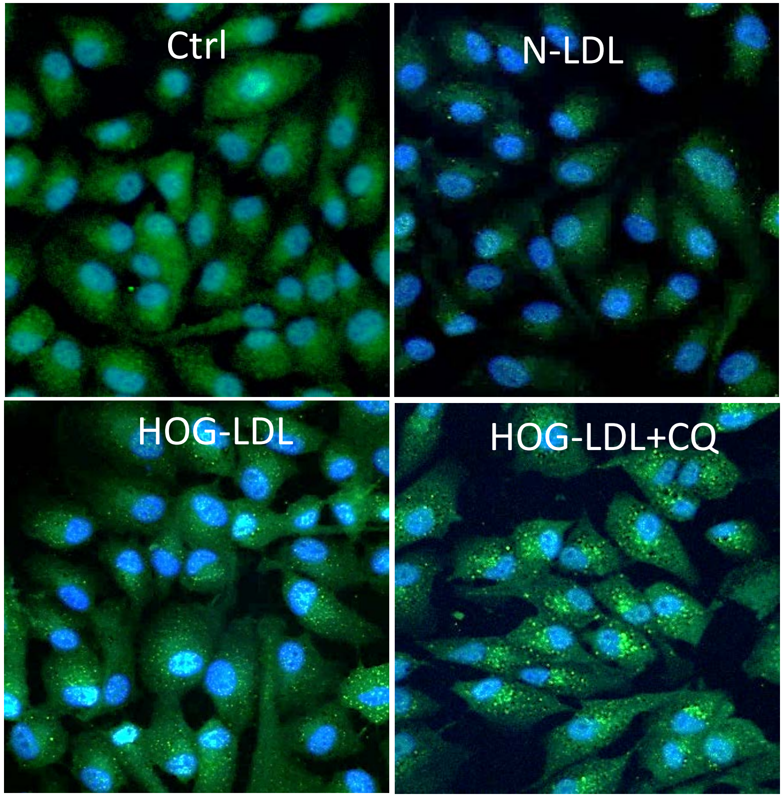

Representative micro-photography of autophagosome formation. Punctate cytoplasmic GFP- LC3B signal is absent in HRCP exposed to SFM or 200mg/l N-LDL (top left and right panels respectively), but present when cells are exposed to HOG-LDL 200mg/l (bottom left), and further enhanced in the presence of HOG-LDL and chloroquine (bottom right).

ESM Fig. 3

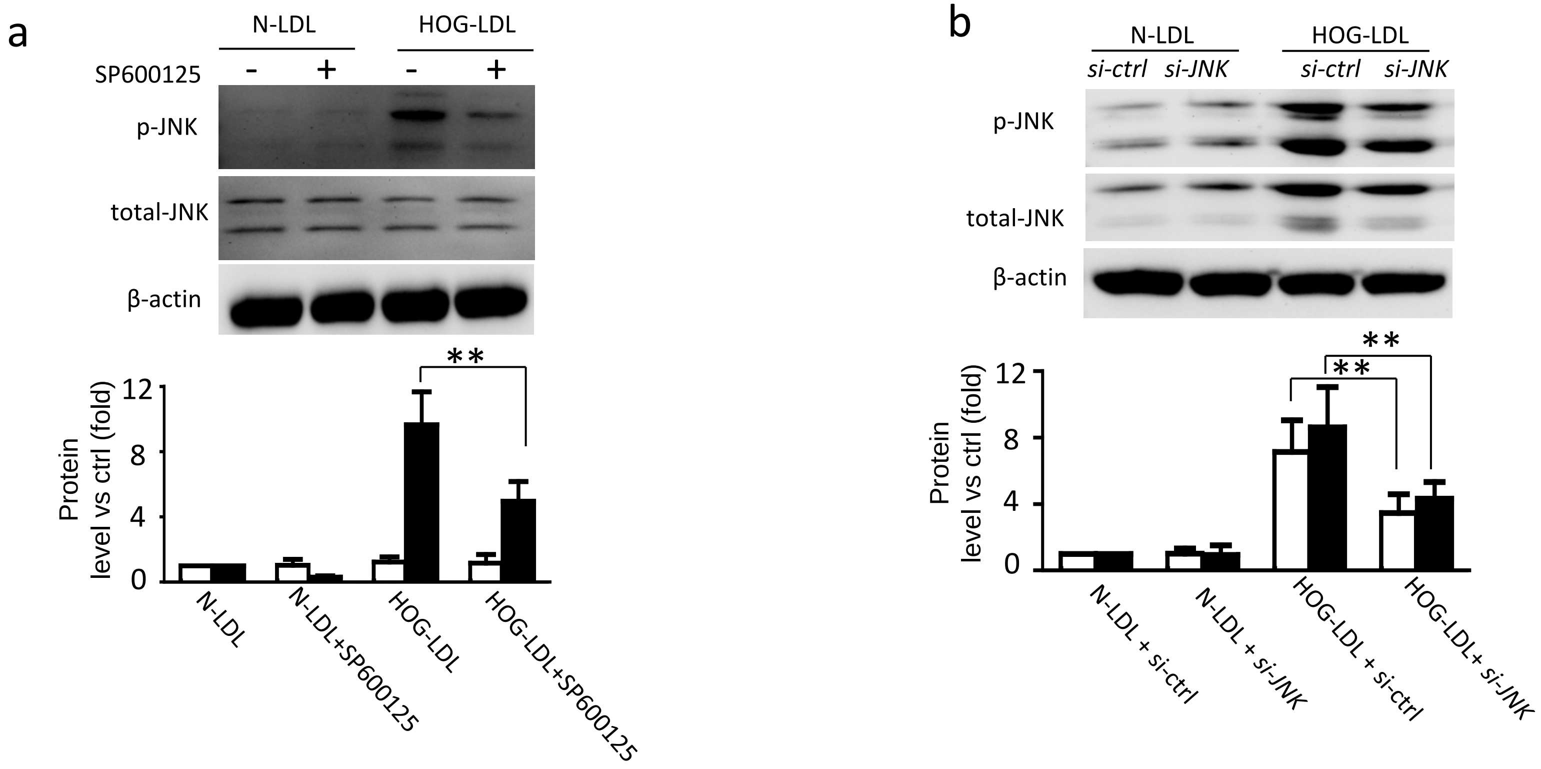

SP600125 or *si-JNK* inhibits JNK activation. Pericytes were pre-treated with **(a)** p-JNK inhibitor SP600125 (10 µmol/l) for 1h, or **(b)** transfected with *si-JNK*/or *si-Ctrl* for 36h, then exposed to N-LDL (200 mg/l) or HOG-LDL (200 mg/l) for 12h. Western blots are shown for total JNK (white bar) and p-JNK (black bar). Means ± S.D., n=3; **p<0.01.

ESM Fig. 4

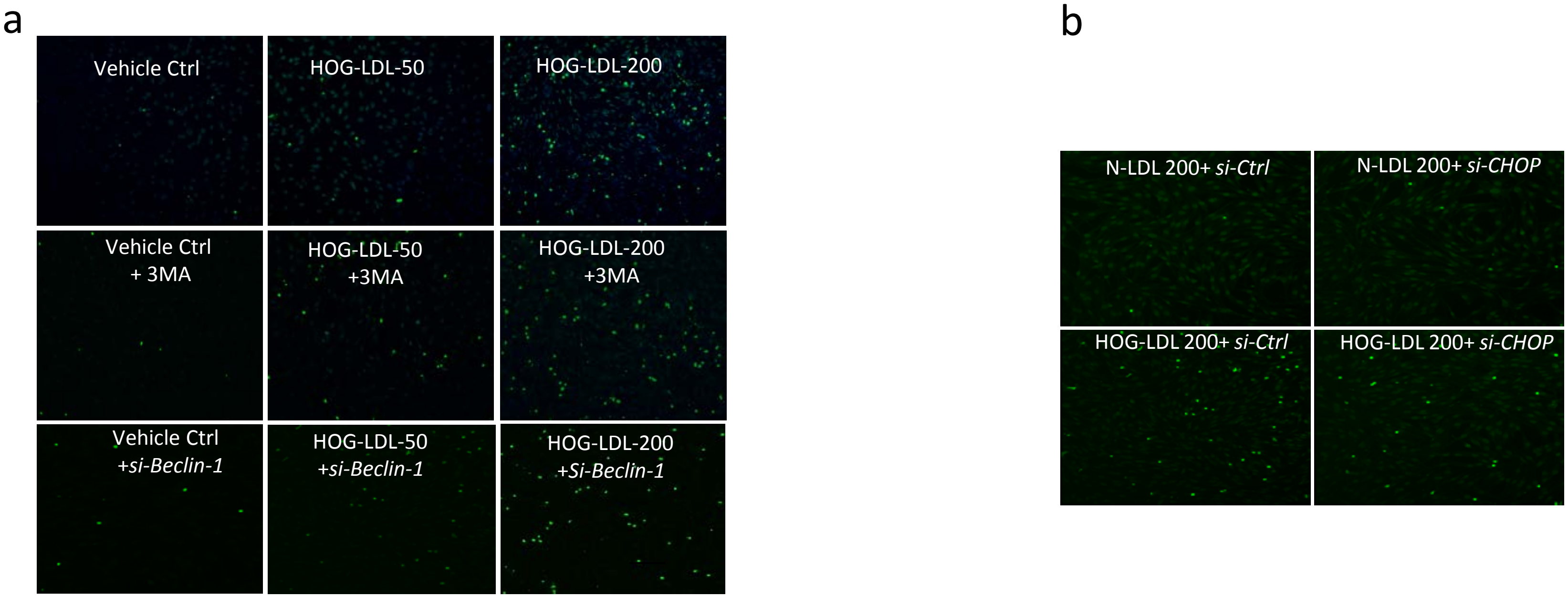

Representative micro-photography of TUNEL staining in HRCP following exposure to N- and HOG-LDL: effects of inhibition of autophagy (a) or CHOP knockout (b). **(a)** To assess effects of inhibiting autophagy, HRCP were pre-treated with 3MA (5 mmol/l, 1h), or transfected with *si-Beclin-1/ si-Ctrl*. **(b)** For CHOP knockout, *si-CHOP* (and *si-ctrl*) for 36h before exposure to N-LDL (200 mg/l) or HOG-LDL (50 or 200 mg/l) for 12h. TUNEL-positive cells were detected by fluorescence microscopy: the figures show representative microphotography of TUNEL staining.
